# Bagging Strategy and Identification of Coloring Mode of ‘Xinqihong’ Pear

**DOI:** 10.3390/ijms23137310

**Published:** 2022-06-30

**Authors:** Jianlong Liu, Hongwei Sun, Xuening Wang, Xin Liu, Hongpeng Xu, Chenglin Liang, Dingli Li, Yingjie Yang, Zhenhua Cui, Jiankun Song, Ran Wang

**Affiliations:** 1 College of Horticulture, Qingdao Agricultural University, Qingdao 266109, China; 201901068@qau.edu.cn (J.L.); shw@stu.qau.edu.cn (H.S.); lbyxxy@126.com (X.W.); lx763849@163.com (X.L.); 20212102044@stu.qau.edu.cn (H.X.); lidingli@qau.edu.cn (D.L.); wuhuaguoyyj@163.com (Y.Y.); zhcui@qau.edu.cn (Z.C.); 200601048@qau.edu.cn (J.S.); 2 Haidu College, Qingdao Agricultural University, Laiyang 265200, China; ljl2015lcl@163.com

**Keywords:** bagging strategy, CRY, light signal, anthocyanin accumulation, red pear

## Abstract

‘Xinqihong’ is a recently selected and well-colored red pear (*Pyrus bretschneideri* Rehd.) cultivar that is popular in the marketplace owing to the bright red color and high quality of the fruit. The red pigmentation is strongly associated with the light signal. However, its responses to bagging treatment and to light exposure after shading are unknown. In this study, the fruit were treated with three types of fruit bags. ’Xinqihong’ fruit colored rapidly in response to light stimulation. A white fruit bag was optimal for bagging of ‘Xinqihong’ fruit. To ensure satisfactory red pigmentation, the fruit required exposure to 30 days of light after bag removal. A transcriptome analysis was conducted to screen light-signal-related genes and identify their possible functions. PbCRY1 activated the promoter of *PbHY5.2* and enhanced its expression. PbHY5.2 activated the promoter activity of *PbUFGT* and induced anthocyanin synthesis, and also showed self-activation characteristics. Both PbCRY2 and PbPHY1 induced anthocyanin accumulation. Thus, blue-light receptors played an important role in anthocyanin synthesis. This study provides a theoretical basis for the bagging cultivation of new varieties of ‘Xinqihong’, and lays a foundation for the study of the mechanisms of red pear fruit coloring in response to light signals.

## 1. Introduction

Bagging is an important aspect of pear (*Pyrus* spp.) production. It is an effective method to reduce the incidence of fruit diseases, insect pests, bird damage, and mechanical damage, as well as to improve fruit skin coloration [1,2]. Bagging enhances the fruit’s sensitivity to light, and the fruit color develops quickly in response to light stimulation. Removal of the fruit bag before harvesting ensures that the best commodity value is attained.

The skin color is an important aesthetic attribute of pear fruit that directly affects consumer appeal [3]. Red-colored European pear (*Pyrus communis* L.) cultivars have been widely cultivated and accepted worldwide. However, in recent years, together with the introduction of local red pear cultivars into breeding programs, red Asian pears have gradually increased in prevalence in the marketplace, and have gained consumers’ acceptance [3,4].

As one of the important components in fruits, anthocyanins are good for health [5,6,7]. In pear cultivars, the red coloration of the fruit skin is the result of anthocyanin accumulation [8]. Anthocyanin biosynthesis is catalyzed by a series of enzymes encoded by structural genes. Anthocyanins are synthesized through the flavonoid pathway, in which phenylalanine ammonia-lyase, chalcone synthase (CHS), chalcone isomerase, flavanone-3-hydroxylase, dihydroflavonol 4-reductase (DFR), anthocyanidin synthase (ANS), and UDP-glucose:flavonoid 3-glucosyltransferase (UFGT) are the crucial enzymes [9,10,11]. The expression abundance of anthocyanin-synthesis-related structural genes has a synergistic effect, which is regulated by various external factors. Light is an essential factor in the biosynthesis of anthocyanins in pears [12,13]. Plants have developed sophisticated photoreceptor systems—such as phytochromes (PHY; red/far-red photoreceptors), cryptochromes, phototropin (CRY; blue/UV-A photoreceptors), and UVR8 (UV-B photoreceptors)—to adapt to variable light radiation [14]. The mechanism of photoinduced anthocyanin biosynthesis in *Arabidopsis* has been demonstrated in studies of photomorphogenesis [15].

The roles of these photoreceptors in regulating anthocyanin accumulation have been confirmed in previous studies. For example, the expression level of *CHS* genes and the anthocyanin content were reduced in *phyA* mutants and *UVR8* mutants [16,17]. In addition, CRY1 regulates the activation of anthocyanin biosynthesis enzymes to induce anthocyanin accumulation under blue light [18]. Many promoter sequences of structural genes associated with anthocyanin synthesis contain *cis*-acting elements involved in light response. Their expression is regulated by light-responsive elements. Anthocyanin biosynthesis is regulated by a MYB–basic/helix-loop-helix–WD repeat (MYB-bHLH-WDR [MBW]) complex [19]. The crucial regulatory genes are also induced by light; for example, PpbHLH64 positively regulates anthocyanin biosynthesis, and is regulated by light at the transcriptional and post-translational levels [20]. The conserved blue-light signal transduction module CRY-COP1-HY5 contributes to anthocyanin biosynthesis induced by blue light in red pears (*Pyrus pyrifolia* (Burm.f.) Nakai). PpHY5 directly bound to the promoters of the anthocyanin biosynthesis genes *PpCHS*, *PpDFR*, *PpANS*, and *PpMYB10*, and activated the transcription of *PpCHS* in a *Nicotiana benthamiana*-based dual-luciferase assay [21,22]. PpBBX16/18/21 and PpHY5 are regulators of light-induced anthocyanin accumulation [8,23]. These studies indicate that HY5 is an important signal integration factor of all known downstream branches of photoreceptors involved in light-induced anthocyanin synthesis in red pears.

Owing to the effect of shading, bagging may lead to failure of the normal coloring of pear fruit. Fruit bags that differ in light transmittance differentially affect fruit color. Fruit enclosed in a white paper bag develops red coloration of greater intensity than fruit enclosed in a double black paper bag or double yellow paper bag [24]. Pre-harvest bag picking is mostly used in production to promote fruit coloring, so as to achieve good-quality attributes. However, different pear cultivars vary in light sensitivity and coloring time. The coloring patterns differ markedly among pear cultivars with different genetic backgrounds. In ‘Xiyanghong’ (*Pyrus bretschneideri* Rehder × *P. communis*), the exposed skin starts to turn red at 57 days after full bloom, and the red coloration is retained until fruit maturity, whereas the skin of bagged fruit is not colored throughout all developmental stages [20]. In one study, the European pear cultivars ‘Starkrimson’ and ‘Red Bartlett’ colored at the beginning of fruit set, but the color intensity decreased with fruit maturation; four different cultivars showed low initial anthocyanin accumulation, and the contents increased during fruit development, but the color intensity also decreased at advanced stages [25]. Consistent results have been reported for red pear cultivars. Many red pear cultivars exhibit fading at an advanced stage of fruit development [26].

The pear cultivar ‘Xinli No. 7’ is widely planted in northern China, but the fruit is prone to fading at an advanced stage of fruit development. The fruit of ‘Xinqihong’—a bud sport of ‘Xinli No. 7’—continue to intensify in color at advanced developmental stages [26]. However, as a newly selected cultivar, the fruit’s response to bagging has not been studied. Therefore, in this study the bagging strategy of ‘Xinqihong’ in production was investigated, and the related mechanism of light regulating anthocyanin accumulation was determined through historical observation, translation analysis, and functional verification of pigment-related genes.

## 2. Results

### 2.1. Effects of Bagging Treatments on the Skin Color of ‘Xinqihong’ Pears

**Figure 1:**
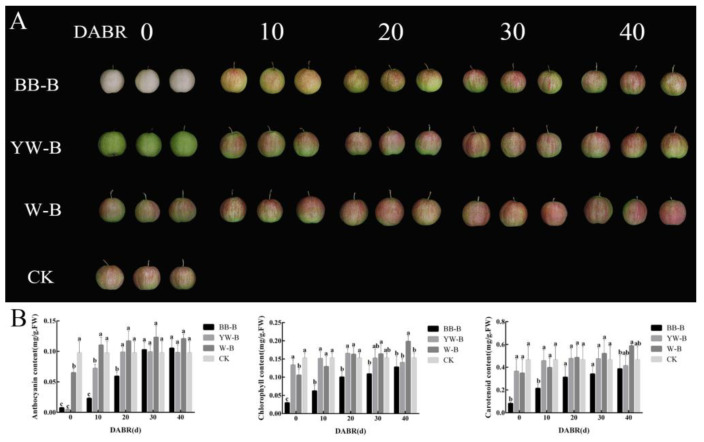
Phenotypes and pigment contents of bagged fruit of ‘Xinqihong’ pears: (**A**) Color phenotype of ‘Xinqihong’ fruit treated by bagging in different types of fruit bag. (**B**) Changes in pigment contents of ‘Xinqihong’ treated with different types of fruit bag. DABR: days after bag removal; BB-B: brown–black-bag-treated fruit; YW-B: yellow–white-bag-treated fruit; W-B: white-bag-treated fruit. Different letters indicate significant differences.

Three types of fruit bags (brown–black bags (BB-B), yellow–white bags (YW-B), and white bags (W-B)) were removed at different time points before harvest. After 40 days of treatment, the fruit appeared white after enclosure with BB-B fruit bags, the fruit were green after treatment with YW-B fruit bags, and fruit bagged with W-B fruit bags showed red coloration. The fruit showed differences in color phenotype reflecting the duration of illumination (Figure 1A). For the different bagging treatments, the anthocyanin, chlorophyll, and carotenoid contents reached the control levels after exposure to light for up to 30 days (Figure 1B).

The fruit’s color parameters significantly changed after removal of the bags (Table 1). After removal of BB-B and YW-B fruit bags for 30 days, the a* value reached that of fruit exposed to normal light, whereas the a* value of fruit treated with W-B fruit bags was significantly higher than that of the other treatments. The brightness L* value of fruit decreased to varying degrees with the deepening of red coloration, whereas the brightness value of treated fruit was higher than that of the controls in the period of the highest red coloration (30 days after bag removal) in all treatments.

### 2.2. Histological Observation of Fruit Skin Tissue

**Figure 2:**
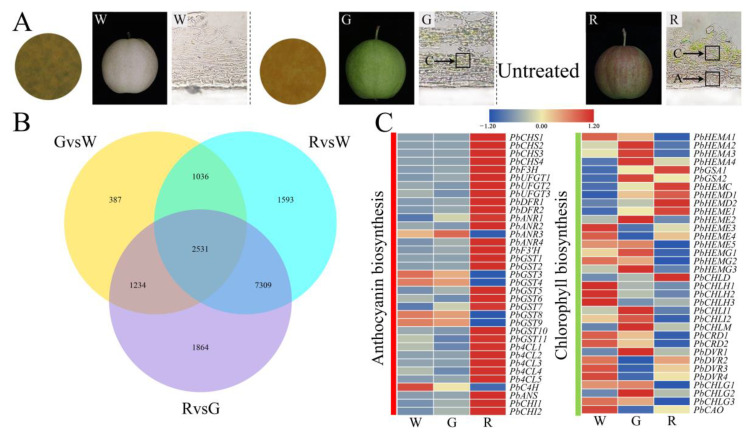
Transverse sections of the pericarp of bagged ‘Xinqihong’ pear fruit, and analysis of differentially expressed genes (DEGs): (**A**) Anatomy of the skin of ‘Xinqihong’ fruit treated with different types of fruit bags. (**B**) Venn diagram of DEGs in the skin of bagged (W and G) and unbagged (R) fruit detected by RNA sequencing. (**C**) Relative expression of candidate genes associated with the anthocyanin and chlorophyll synthesis pathways. W: Brown–black-bag-treated fruit; G: yellow–white-bag-treated fruit; R: fruit not bagged.

The pigmentation of the ‘Xinqihong’ fruit skin was observed with a microscope. After BB-B fruit-bagging treatment, the fruit were white and the skin cells lacked chlorophyll and anthocyanins. The fruit treated with YW-B fruit bags were green, and chlorophyll-enriched cells were located in the outermost 4–6 layers of cells under the epidermis. The color of the fruit without bagging treatment was normal. The cells in 1–3 layers under the epidermis were enriched with anthocyanins, and the lower 4–6 cell layers were rich in chlorophyll. Anthocyanins were located in the upper layer of the chlorophyll-containing cells, and the fruit was red (Figure 2A).

### 2.3. Expression of Genes Associated with Anthocyanin, Chlorophyll Biosynthesis, and Light Response in Fruit Skins of Different Colors

To explore the coloration mechanism of ‘Xinqihong’, a transcriptome analysis was conducted using W (brown–black-bag-treated), G (yellow–white-bag-treated), and R (non-bagged) fruit. Differentially expressed genes (DEGs) were identified in the comparison of R and G fruit, and no overlap of the genes identified in the comparison of G and W fruit was detected. After removing the DEGs from the comparison between R and W fruit, 7309 DEGs were retained (Figure 2B). These genes were selected as candidate genes associated with pericarp coloring of ‘Xinqihong’ for further analysis. The anthocyanin biosynthesis genes *PbCHS*, *PbF3H*, *PbUFGT*, *PbDFR*, *PbANR*, *PbF3′H*, *PbGST*, *Pb4CL*, *PbC4H*, *PbANS*, and *PbCHI* showed similar trends to anthocyanin content in the fruit skin. These genes were highly expressed in the skin of red fruit. Genes associated with chlorophyll synthesis were highly expressed in white and green fruit, and their expression was decreased in red fruit, except for *PbCHLD*, *PbHEMC*, *PbHEMD1*, and *PbHEMD2* (Figure 2C and Table S1).

**Figure 3:**
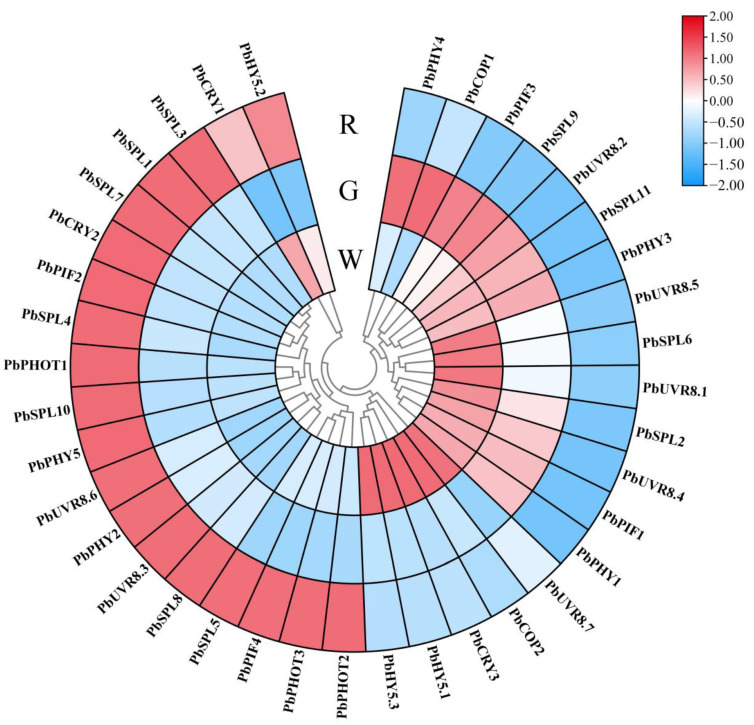
Expression patterns of genes involved in response to the light signal: W: brown–black-bag-treated fruit; G: yellow–white-bag-treated fruit; R: fruit not bagged. Bar: normalized FPKM. The unrooted dendrogram was generated by hierarchical clustering.

Given the influence of bagging on the light signal, the light-signal-related genes were screened. The light receptor genes *PbPHYs* (600–750 nm) and *PbCRYs* (320–500 nm) were significantly upregulated and highly expressed in red skin, whereas the UV-B receptor genes *PbUVRs* were upregulated in white skin. The crucial light-signal-regulating genes *PbHY5s* were highly expressed in red skin induced by light. Hierarchical clustering revealed that *PbCRY1* and *PbHY5.2* were detected in the same dataset (Figure 3 and Table S1).

### 2.4. Expression Patterns of Anthocyanin Synthesis and Red-/Blue-Light Receptor Genes

**Figure 4:**
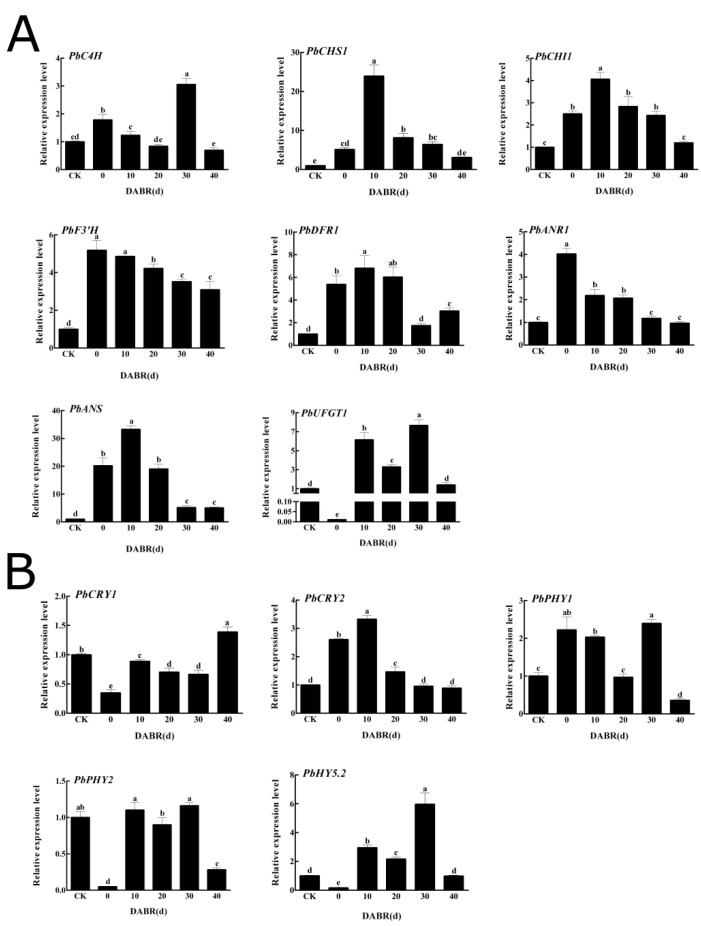
Relative expression levels of genes associated with light response and anthocyanin synthesis in the skin of ‘Xinqihong’ pear fruit after bag removal: (**A**) Expression levels of anthocyanin-synthesis-related genes. (**B**) Expression levels of light-response-related genes. DABR: days after bag removal. Different letters indicate significant differences.

Quantitative real-time PCR (qRT-PCR) analysis was used to verify the differential expression of the screened genes. The expression trend for anthocyanin synthesis genes increased to varying degrees after exposure to light. The expression level of *PbUFGT* in the skin after exposure to light for 10 days was more than 567-fold higher than that in the skin without light exposure. The expression levels of anthocyanin synthesis genes induced by light were significantly higher than those of the controls (Figure 4A). Analysis of the main red- and blue-light receptors *PbCRYs* and *PbPHYs* showed that *PbCRY1*, *PbCRY2*, *PbPHY1*, and *PbPHY2* were significantly affected by light exposure, and were significantly upregulated after exposure to light. In addition, *PbHY5.2* was highly expressed under light induction (Figure 4B).

### 2.5. Verification of Transient Expression of Light-Response-Related Genes in Fruit Skin

**Figure 5:**
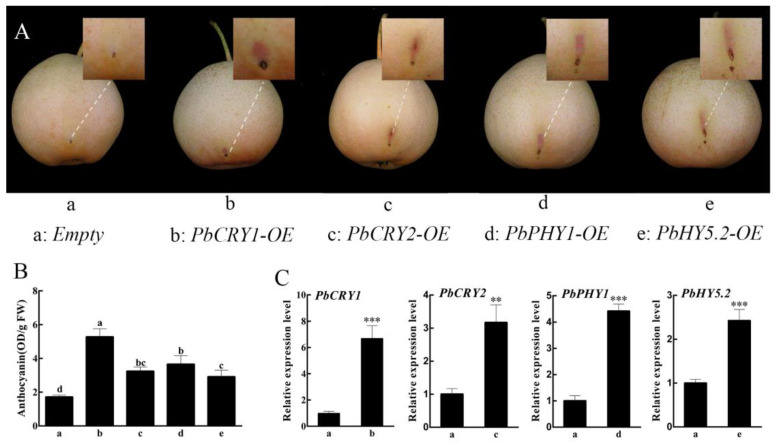
Transient overexpression of PbCRY1, PbPHY1, PbHY5.2, and PbCRY2 in the skin of pear fruit: (**A**) PbCRY1, PbPHY1, PbHY5.2, and PbCRY2 overexpression in ‘Qindaohong’ pear. (**B**) Anthocyanin content in the skin; FW: fresh weight. (**C**) Relative expression levels of *PbCRY1*, *PbPHY1*, *PbHY5.2*, and *PbCRY2* detected by qRT-PCR analysis. Data are the mean ± S.E. of three replicates. Different letters above the bars indicate significant differences (Duncan’s multiple range test *p* < 0.05). Asterisks above the bars indicate significant differences in expression level (Student’s *t*-test: ** *p* < 0.01, *** *p* < 0.001).

To further verify the role of photoresponsive genes in pear anthocyanin synthesis, *PbPHY2*, *PbCRY1*, *PbHY5.2*, *PbPHY1*, and *PbCRY2* were transiently overexpressed in ‘Qindaohong’ pear fruit. After 5 days of exposure to natural light, the anthocyanin biosynthesis genes *PbCRY1*, *PbPHY1*, *PbHY5.2*, and *PbCRY2* were expressed in the skin around the injection site. No anthocyanin accumulation was detected after injection with empty agrobacteria or after transient overexpression of *PbPHY2* (Figure 5A and Figure S1). The anthocyanin contents and relative gene expression levels were analyzed. PbCRY1, PbPHY1, PbHY5.2, and PbCRY2 caused the accumulation of anthocyanin (Figure 5B), and the genes were significantly upregulated at the injection site (Figure 5C).

### 2.6. PbCRY1, PbCRY2, and PbPHY1 Modulate Expression of Target Genes and Anthocyanin Synthesis via PbHY5.2

**Figure 6:**
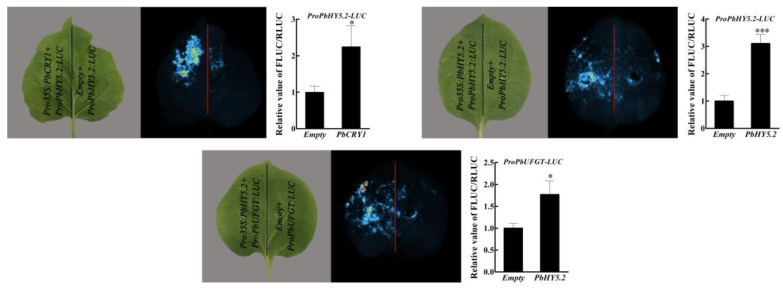
Detection and live imaging of transcriptional activation activity: a representative image of a *Nicotiana benthamiana* leaf 3 days after infiltration is shown. Quantitative analysis on the right corresponds to live imaging. The experiments were independently repeated three times and yielded similar results. Data are the mean ± SD from three replicates (*n* = 3). Asterisks indicate a significant difference (two-tailed Student’s *t*-test: * *p* < 0.05, *** *p* < 0.001).

To investigate whether the *PbCRY* and *PbPHY* genes function in the anthocyanin synthesis pathway, a dual-luciferase assay was performed. The protein PbCRY2 was able to activate expression of *PbHY5.2*, while PbHY5.2 could activate expression of *PbUFGT*. In addition, PbHY5.2 showed self-activation (Figure 6). *PbCRY1* and *PbPHY1* were not able to activate expression of *PbHY5.2*, and may therefore affect anthocyanin synthesis through other pathways (Figure S2).

## 3. Discussion

Fruit bagging during cultivation protects the fruit from infection by pathogens and improves the smoothness of the fruit surface [27,28,29]. For conventional cultivars, bagging is usually applied to immature fruit until the fruit has matured. However, bagging may cause poor fruit coloring because of the shading of light in red pear cultivars. Therefore, a satisfactory appearance of the fruit is often ensured by removing the bags before fruit ripening in crop production.

‘Xinqihong’ is a red pear cultivar with excellent fruit quality. Three types of fruit bags were used for the present treatments, and the bags were removed at different developmental stages to observe the degree of coloring. Bagging treatment significantly increased the L* value (brightness) of the fruit surface. The gene expression analysis also showed that the anthocyanin synthesis gene showed a significant upward trend within 30 days after removing the fruit bag. Therefore, the fruit color began to recover after bag removal with the increase in the duration of light exposure. The W-B treatment had the least impact on the fruit color, which returned to the normal color within 20 days. The fruits treated with BB-B and YW-B bags had returned to the normal fruit color at 30 days after exposure to light. The anthocyanin, chlorophyll, and carotenoid contents reached the control levels after exposure to light for up to 30 days. Therefore, it is suggested that, regardless of the fruit bag type used for ‘Xinqihong’ pears, the fruit coloring should not be affected provided that the bag is removed 30 days before harvest. Previous studies have reported the differences in coloring patterns between pear cultivars [30]. The fruit of ‘Xinli 7’ is colored at an early stage of fruit development, and fades at an advanced stage. Unlike apples, some colored pear cultivars attain peak coloration well before the fruit are ripe, and pre-harvest color loss reduces the crop quality. The European pear cultivars ‘Starkrimson’ and ‘Red Bartlett’ also belong to this group of cultivars [31,32], whereas ‘Xinqihong’ fruit have good coloring ability at advanced stages of development. Through bagging treatment, the coloring ability of ‘Xinqihong’ fruit was shown to be inseparable from light exposure.

In pears, light is essential for anthocyanin biosynthesis [4]. Therefore, transcriptional analysis was used to explore the mechanisms by which light regulates the fruit coloring of ‘Xinqihong’. Anthocyanin synthesis genes were upregulated in red skin, and a large number of light-responsive genes were screened. *PbUFGT*, a crucial gene in anthocyanin synthesis, was highly expressed in response to light. *UFGT* is a critical gene involved in anthocyanin biosynthesis in red-skinned pear cultivars, and variation in the expression of *PbUFGT* leads to coloration differences consistent with light-response patterns [33]. In a previous study, it was revealed that PbUFGT was upregulated through activation by the light-response element PbHY5 [30]. Therefore, *PbHY5* genes were screened among the DEGs. PbHY5.2 was highly expressed in red skin, where it was significantly higher than that in W and G fruit. The present results further confirm that PbHY5.2 can induce anthocyanin synthesis and activate the promoter of PbUFGT. In previous studies, HY5 was shown to have self-activation activity. The PyHY5 protein binds to the promoters of PyWD40 and PyMYB10, and regulates their expression in red ‘Yunhongli No. 1’ pears [22]. In the present study, PbHY5.2 could also activate its own promoter. In the process of light-induced anthocyanin synthesis in red pears, HY5 is an important signal integration factor of downstream branches of photoreceptors. Three types of photoreceptors are known in plants: the blue photoreceptor cryptochrome (CRY) [34,35], the blue photoreceptor phototropin (PHOT) [36,37,38], and the red/far-red photoreceptor phytochrome (PHY) [39]. These receptors may participate in light morphogenesis through HY5. The presence of HY5 and CRY transcription factors among the early light-responsive genes indicated the pivotal role played by light—especially blue light—in the biological changes that occurred after bag removal [31]. Therefore, three blue-light receptors (*PbCRY* genes) were screened from among the DEGs. PbCRY1 and PbCRY2 were highly expressed in the skin of red fruit. Further qRT-PCR verification showed that PbCRY1 and PbCRY2 were highly expressed in response to light exposure. The transient overexpression of PbCRY1 and PbCRY2 confirmed that they are capable of stimulating anthocyanin synthesis. However, only PbCRY1 was able to activate the PbHY5.2 promoter, whereas PbCRY2 did not. Previous studies have reported the involvement of an indirect action module—CRY–COP–HY5—in photomorphogenesis [21,22].

In *Arabidopsis*, CRY has also been shown to induce CHS expression and promote anthocyanin synthesis [40]. Therefore, induction of anthocyanin synthesis by PbCRY2 may be mediated by its regulation of structural genes. Through the expression pattern analysis, the expression pattern of *PbPHY1* was observed to be similar to that of *PbCRY2*, which responded to light signals immediately. However, the expression abundance of *PbPHY1* did not increase with the increase in the duration of light exposure. *PbPHY1* could induce anthocyanin synthesis, but could not activate the *PbHY5.2* promoter. PbPHY2 could not induce anthocyanin synthesis.

## 4. Materials and Methods

### 4.1. Plant Materials

**Figure 7:**
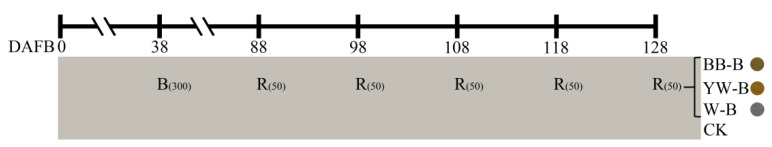
Schematic illustration of the experimental treatment: Samples were collected 128 days after full bloom. B: bagging, R: removal of the bags, DAFB: days after full bloom, BB-B: brown–black bag, YW-B: yellow–white bag, W-B: white bag, CK: no bag.

Samples of healthy and uniform 3-year-old ‘Xinqihong’ pear plants that had been grafted onto *P*. *betulifolia* Bunge rootstocks were collected at the Jiaodong Peninsula Regional Experimental Park (37.52° N, 120.25° E). This region experiences a warm–temperate continental monsoon climate, with average annual precipitation of 672.5 mm and an average annual temperature of 12.6 °C. Fruit with consistent growth and no shading were selected 38 days after flowering and bagged in either a brown–black bag, a yellow–white bag, or a white bag (Table S2). The fruit bags were removed at 88, 98, 108, 118, and 128 days after full bloom (DAFB) (i.e., 40, 30, 20, 10, and 0 days before harvest, respectively). The controls were non-bagged fruit (Figure 7). The fruit were harvested at commercial maturity (128 DAFB). For each sample, the skin of 10 fruit was removed, immediately frozen in liquid nitrogen, and stored at −80 °C for total RNA isolation and measurement of pigment content.

### 4.2. Fruit Color Measurement

The color of the fruit skin was determined using a portable color difference meter (CR-400; Konica Minolta, Tokyo, Japan). The L*, a*, and b* values were measured at the equator of the fruit, where L* is the brightness of the color; a* is the red–green coordinate value, which varies from −80 to 100 from green to red (with a higher absolute value indicating a deeper red or green color); and b* is the blue–yellow coordinate value, which varies from −80 (blue) to 70 (yellow) (with a larger absolute value indicating a darker color).

### 4.3. Measurement of Anthocyanin, Chlorophyll, and Carotenoid Contents in Fruit Skin

Approximately 1 g of fruit skin was ground to a fine powder in liquid nitrogen and extracted with 5 mL of extraction solution (1% HCl in methanol) at 4 °C for 12 h. After centrifugation at 12,000× *g* for 20 min, the supernatant was transferred to a clean tube, and the absorbance at 510 nm was measured with a spectrophotometer (UV1800; Meipuda, Shanghai, China). The anthocyanin content was calculated using the equation *C*_a_ = 1000 × *A* × *V*/(*a* × *b* × *W*), where *C*_a_ is the total anthocyanin content (mg/g), *A* is the absorbance value, *V* is the extraction solution volume, *a* is the absorptivity of anthocyanin (0.0775), *b* is the thickness of the colorimetric ware, and *W* is the fresh weight of the fruit skin.

Anthocyanins were extracted from the fruit skin using methanol:HCl (99:1, *v/v*), as described by Bai et al. [41]. The absorbance (optical density; OD) at 530, 620, and 650 nm was assessed using a spectrophotometer (DU800, Beckman Coulter; https://www.beckmancoulter.com/, accessed on 26 April 2022). Anthocyanin content (*A*) was represented by the normalized OD value, calculated as *A* = [(*A*_530_ − *A*_650_) − 0.2 × (*A*_650_ − *A*_620_)] / 0.1. Chlorophyll and carotenoid contents were calculated as previously described [42,43,44]. Data for three replicates of each sample were averaged.

### 4.4. Observation of Frozen Sections of Pericarp

White fruit (W), green fruit (G), and red fruit (R) of ‘Xinqihong’ sampled at 128 DAFB were washed. W and G comprised pure-colored fruits. The skin of W and G fruits was peeled. The CK was red fruit skin. The skin at the most darkly colored position was sampled to observe and photograph transverse sections of the skin. Sections (10 μm thickness) of the darkest peel were cut with a Microm HM 525 cryostat and observed under a Leica DM2500 fluorescence microscope equipped with a 20× objective lens.

### 4.5. RNA Extraction and Transcriptome Sequencing

For library construction, 1 μg of RNA per sample was used as the input material. The PCR products were purified (using the AMPure XP system), and library quality was assessed with a 2100 Bioanalyzer system (Agilent, Santa Clara, CA, USA). Clean reads were filtered from the raw data by removing reads containing the adapter or poly-N together with low-quality reads. Reference genome and gene model annotation files were downloaded from the genome website (https://www.ncbi.nlm.nih.gov/genome/12793, accessed on 5 June 2022). An index of the reference genome was constructed using Hisat2 v2.0.5 (http://daehwankimlab.github.io/hisat2/, accessed on 11 February 2022), which was also used to align paired-end clean reads to the reference genome. The mapped reads of each sample were assembled with StringTie v1.3.3b (https://ccb.jhu.edu/software/stringtie/, accessed on 15 February 2022) using a reference-based approach. FeatureCounts v1.5.0-p3 was used to count the number of reads mapped to each gene. The fragments per kilobase of transcript per million mapped reads (FPKM) value for each gene was calculated based on the length of the gene and the number of reads mapped to the gene. Three RNA sequencing libraries for W, G, and R fruit were constructed.

### 4.6. qRT-PCR Analysis

The cDNA templates were reverse-transcribed using total RNA extracted from the skin of five samples of ‘Xinqihong’ fruit after removal of the BB-B bags at different time points. Amplification by qRT-PCR was conducted as follows: 95 °C for 5 min, followed by 45 cycles of 95 °C for 15 s, 60 °C for 30 s, and 72 °C for 30 s using a Roche 480 Real-Time PCR system (Roche, Basil, Switzerland) in the standard mode with the FastStart Essential DNA Green Master Kit. All reactions were performed in triplicate at a volume of 20 µL, containing 2 µL of 10-fold-diluted cDNA. The pear *Actin* gene was used as an internal control. The primers used are listed in Table S2.

### 4.7. Transient Transformation Analysis in Pear Fruit

For the transient overexpression assay, mature fruit of ‘Qindaohong’ pear were used. The ‘Qindaohong’ pear bears fruit that are blushed on the side exposed to the sun, which indicates the presence of an intact anthocyanin biosynthesis pathway in this cultivar and, thus, makes it a suitable material for observation of the induction of coloration. The fruit were infiltrated with *Agrobacterium tumefaciens* strain EHA105 harboring the pGreenII 0029 62-SK-PbCRY1, pGreenII 0029 62-SK-PbPHY1, pGreenII 0029 62-SK-PbHY5.2, or pGreenII 0029 62-SK-PbCRY2 vectors via an injection syringe, in accordance with a previously described protocol, with slight modifications [45]. Briefly, *A*. *tumefaciens* was grown to saturation in Luria–Bertani medium. After centrifugation at 13,400× *g*, the pellet was resuspended in the infection solution (10 mM MgCl_2_, 10 mM MES, and 150 mM acetosyringone), and the mixture was incubated at room temperature for 1 h. After infiltration, the fruit were kept in the dark for 1 d and then treated with continuous natural light for 5 days. The empty pGreenII 0029 62-SK vector was used as the negative control (Empty). After images were captured, the fruit peel surrounding the injection sites was collected, immediately frozen in liquid nitrogen, and stored at −80 °C until use.

### 4.8. Dual-Luciferase Reporter System Assays

Dual-luciferase assays were performed with *N*. *benthamiana* leaves as previously reported [46]. The full-length coding sequence of each gene was cloned into the pGreenII 0029 62-SK vector, and the promoter sequence of the *cis* gene was inserted into the pGreenII 0800-LUC vector. Both constructs were individually transformed into *A. tumefaciens* strain EHA105 (harboring the pSoup vector). The firefly luciferase and Renilla luciferase activities were analyzed 72 h after infiltration using the Dual-Luciferase Reporter Assay System (E710, Promega, Madison, USA) with a modular luminometer (GloMax96, Promega). Both luciferase activities were analyzed in three independent experiments with at least six biological replicates for each assay. The primers used are listed in Table S2.

### 4.9. Statistical Analysis

Statistical analysis was performed using IBM SPSS Statistics 23.0 (IBM Corporation, Armonk, NY, USA). Values are presented as the mean ± SD of at least three independent biological replicates. Data were analyzed with Duncan’s multiple range test or Student’s *t*-test. The probability level *p* < 0.05 was considered to be significant.

## 5. Conclusions

**Figure 8:**
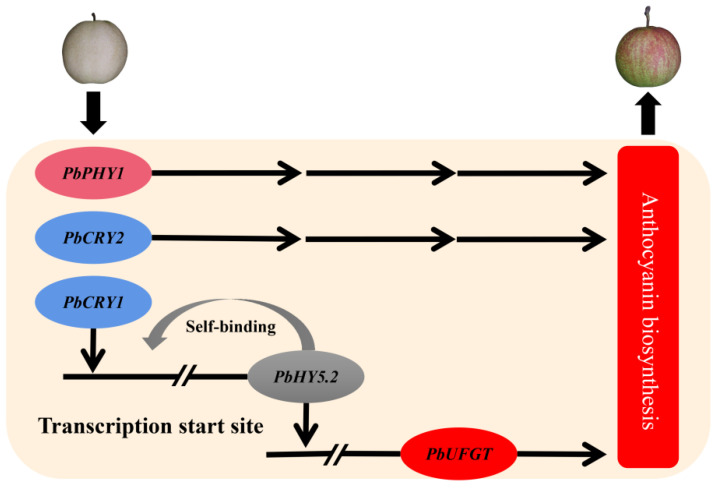
Model for the regulation of light-induced anthocyanin biosynthesis in fruit of ‘Xinqihong’ pear.

In this study, the W-B fruit bag was the optimal choice for bagging treatment of ‘Xinqihong’ pear fruit. To ensure that the fruit develop a red coloration, they require exposure to light for 30 days after removal of the bag. Bagging treatments increase the brightness of the fruit surface. The present and previous findings indicate that blue-light receptors play an important role in anthocyanin synthesis [31]. PbCRY1 activates the promoter of *PbHY5.2* and enhances its expression in red pears. The protein PbHY5.2 activates the promoter activity of *PbUFGT* and induces anthocyanin synthesis, and also has self-activation characteristics. In addition, both PbCRY2 and PbPHY1 induce anthocyanin synthesis (Figure 8). This study provides a theoretical basis for the bagging treatment of the new cultivar ‘Xinqihong’, and lays a foundation for further study of the mechanisms of red pear fruit coloring in response to light signals.

## Figures and Tables

**Table 1:** Color parameters among ‘Xinqihong’ pear fruit bagged with three types of fruit bag for different periods.

Treatment	DABR (d)	Parameters		
		L*	a*	b*
BB-B	0	66.19 ± 2.47 ^a^	−9.55 ± 0.53 ^a^	28.76 ± 1.16 ^a^
	10	50.66 ± 2.37 ^bc^	1.24 ± 1.07 ^b^	22.48 ± 1.13 ^bc^
	20	47.41 ± 1.51 ^c^	4.51 ± 0.47 ^c^	20.11 ± 1.30 ^cd^
	30	42.77 ± 1.63 ^d^	9.07 ± 0.19 ^fg^	16.87 ± 0.73 ^ef^
	40	38.16 ± 3.05 ^fg^	9.19 ± 0.30 ^fg^	15.71 ± 0.62 ^f^
YW-B	0	52.78 ± 1.88 ^b^	−13.69 ± 1.19 ^e^	24.31 ± 3.54 ^b^
	10	41.38 ± 1.36 ^def^	6.46 ± 1.16 ^d^	19.09 ± 0.50 ^de^
	20	42.41 ± 1.40 ^de^	8.79 ± 0.75 ^fg^	17.14 ± 1.57 ^ef^
	30	40.16 ± 1.45 ^def^	8.94 ± 1.09 ^fg^	17.09 ± 0.61 ^ef^
	40	34.96 ± 1.84 ^g^	9.44 ± 2.20 ^fgh^	15.75 ± 0.28 ^f^
W-B	0	40.14 ± 2.54 ^def^	5.33 ± 0.50 ^cd^	17.13 ± 1.71 ^ef^
	10	39.01 ± 3.27 ^def^	10.69 ± 0.86 ^ghi^	16.07 ± 1.75 ^f^
	20	41.22 ± 1.92 ^def^	11.04 ± 0.62 ^ghi^	16.60 ± 0.98 ^ef^
	30	39.17 ± 0.99 ^def^	10.58 ± 1.48 ^ghi^	17.19 ± 1.68 ^ef^
	40	38.76 ± 2.54 ^ef^	11.51 ± 0.87 ^i^	16.01 ± 1.64 ^f^
CK	-	38.30 ± 1.19 ^fg^	8.57 ± 0.79 ^f^	15.68 ± 0.88 ^f^

Data are the mean ± standard error (*n* = 5). Different lowercase letters within a column are statistically significant at *p* < 0.05 (Duncan’s test). L*, a*, and b* represent different fruit color parameters. BB-B: brown–black bag, YW-B: yellow–white bag, W-B: white bag, CK: no bag.

## Data Availability

Data are contained within the article or the Supplementary Materials.

## Supplementary Materials

The following supporting information can be downloaded at: https://www.mdpi.com/article/10.3390/ijms23137310/s1.

## References

[1] Frank D.L. (2018). Evaluation of fruit bagging as a pest management option for direct pests of apple. Insects.

[2] Sharma R.R., Reddy S.V.R., Jhalegar M.J. (2014). Pre-harvest fruit bagging: A useful approach for plant protection and improved post-harvest fruit quality—A review. J. Hortic. Sci. Biotech..

[3] Zhang B.B., Guo J.Y., Ma R.J., Cai Z.X., Yan J., Zhang C.H. (2015). Relationship between the bagging microenvironment and fruit quality in ‘Guibao’ peach [*Prunus persica* (L.) Batsch]. J. Hortic. Sci. Biotech..

[4] Sun S., Xin L., Gao H., Wang J., Li P. (2014). Response of phenolic compounds in ‘Golden Delicious’ and ‘Red Delicious’ apples peel to fruit bagging and subsequent sunlight re-exposure. Sci. Hortic..

[5] Farzaneh V., Carvalho I.S. (2015). A review of the health benefit potentials of herbal plant infusions and their mechanism of actions. Ind. Crops Prod..

[6] Farzaneh V., Gominho J., Pereira H., Carvalho I.S. (2018). Screening of the antioxidant and enzyme inhibition potentials of portuguese *Pimpinella anisum L*. seeds by GC-MS. Food Anal. Methods.

[7] Farzaneh V., Carvalho I.S. (2017). Modelling of microwave assisted extraction (MAE) of anthocyanins (TMA). J. Appl. Res. Med. Aromat. Plants.

[8] Bai S., Tao R., Tang Y., Yin L., Ma Y., Ni J., Yan X., Yang Q.S., Wu Z., Zeng Y. (2019). BBX16, a B-box protein, positively regulates light-induced anthocyanin accumulation by activating *MYB10* in red pear. Plant Biotechnol. J..

[9] Qian M., Yu B., Li X., Sun Y., Zhang D., Teng Y. (2014). Isolation and expression analysis of anthocyanin biosynthesis genes from the red Chinese sand pear, *Pyrus pyrifolia* Nakai cv. Mantianhong, in response to methyl jasmonate treatment and UV-B/VIS conditions. Plant Mol. Biol. Rep..

[10] Jin W., Wang H., Li M., Wang J., Yang Y., Zhang X., Yan G., Zhang H., Liu J., Zhang K. (2016). The R2R3 MYB transcription factor *PavMYB10.1* involves in anthocyanin biosynthesis and determines fruit skin colour in sweet cherry (*Prunus avium* L.). Plant Biotechnol. J..

[11] Liu H., Liu Z., Wu Y., Zheng L., Zhang G. (2021). Regulatory mechanisms of anthocyanin biosynthesis in apple and pear. Int. J. Mol. Sci..

[12] Tao R., Yu W., Gao Y., Ni J., Yin L., Zhang X., Li H., Wang D., Bai S., Teng Y. (2020). Light-induced basic/helix-loop-helix64 enhances anthocyanin biosynthesis and undergoes CONSTITUTIVELY PHOTOMORPHOGENIC1-mediated degradation in pear. Plant Physiol..

[13] Sun Y., Qian M., Wu R., Niu Q., Teng Y., Zhang D. (2014). Postharvest pigmentation in red Chinese sand pears (*Pyrus pyrifolia* Nakai) in response to optimum light and temperature. Postharvest Biol. Technol..

[14] Paik I., Huq E. (2019). Plant photoreceptors: Multi-functional sensory proteins and their signaling networks. Semin. Cell Dev. Biol..

[15] Maier A., Schrader A., Kokkelink L., Falke C., Welter B., Iniesto E., Rubio V., Uhrig J.F., Hülskamp M., Hoecker U. (2013). Light and the E3 ubiquitin ligase COP1/SPA control the protein stability of the MYB transcription factors PAP1 and PAP2 involved in anthocyanin accumulation in Arabidopsis. Plant J..

[16] Kunkel T., Neuhaus G., Batschauer A., Chua N.-H., Schäfer E. (1996). Functional analysis of yeast-derived phytochrome A and B phycocyanobilin adducts. Plant J..

[17] Kliebenstein D.J., Lim J.E., Landry L.G., Last R.L. (2002). Arabidopsis *UVR8* regulates ultraviolet-B signal transduction and tolerance and contains sequence similarity to human *Regulator of Chromatin Condensation 1*. Plant Physiol..

[18] Ahmad M., Lin C., Cashmore A.R. (1995). Mutations throughout an *Arabidopsis* blue-light photoreceptor impair blue-light-responsive anthocyanin accumulation and inhibition of hypocotyl elongation. Plant J..

[19] Allan A.C., Hellens R.P., Laing W.A. (2008). MYB transcription factors that colour our fruit. Trends Plant Sci..

[20] Wei T., Wang C., Qi T., An Z., Wu M., Qu L., Li J., Wen Y., Shi Q., Zhai R. (2020). Effect of natural light on the phenolic compounds contents and coloration in the peel of ‘Xiyanghong’ (*Pyrus bretschneideri*×*Pyrus communis*). Sci. Hortic..

[21] Tao R., Bai S., Ni J., Yang Q., Zhao Y., Teng Y. (2018). The blue light signal transduction pathway is involved in anthocyanin accumulation in ‘Red Zaosu’ pear. Planta.

[22] Wang Y., Zhang X., Zhao Y., Yang J., He Y., Li G., Ma W., Huang X., Su J. (2020). Transcription factor PyHY5 binds to the promoters of *PyWD40* and *PyMYB10* and regulates its expression in red pear ‘Yunhongli No. 1’. Plant Physiol. Biochem..

[23] Bai S., Tao R., Yin L., Ni J., Yang Q., Yan X., Yang F., Guo X., Li H., Teng Y. (2019). Two B-box proteins, PpBBX18 and PpBBX21, antagonistically regulate anthocyanin biosynthesis via competitive association with *Pyrus pyrifolia* ELONGATED HYPOCOTYL 5 in the peel of pear fruit. Plant J..

[24] Kim Y.K., Kang S.S., Choi J.J., Park K.S., Won K.H., Lee H.C., Han T.H. (2014). The effect of several paper bags on fruit skin coloration of red skin European pear ‘Kalle’. Korean J. Hortic. Sci. Technol..

[25] Yang Y.-N., Yao G.-F., Zheng D., Zhang S.-L., Wang C., Zhang M.-Y., Wu J. (2015). Expression differences of anthocyanin biosynthesis genes reveal regulation patterns for red pear coloration. Plant Cell Rep..

[26] Farzaneh V., Ghodsvali A., Bakhshabadi H., Dolatabadi Z., Farzaneh F., Carvalho I.S., Sarabandi K. (2018). Screening of the alterations in qualitative characteristics of grape under the impacts of storage and harvest times using artificial neural network. Evol. Syst. Ger..

[27] Liu J., Deng Z., Sun H., Song J., Li D., Zhang S., Wang R. (2021). Differences in anthocyanin accumulation patterns and related gene expression in two varieties of red pear. Plants.

[28] Wan Y., Hou Q.-R., Wen Y., Wang L., Lu Q. (2016). Bagging technology reduces pesticide residues in table grapes. J. Am. Pomol. Soc..

[29] Chen B., Mao J., Huang B., Mi B., Liu Y., Hu Z., Ma Z. (2017). Effect of bagging and time of harvest on fruit quality of ‘Red Fuji’ apple in high altitude area in China. Fruits.

[30] Liu H., Su J., Zhu Y., Yao G., Allan A.C., Ampomah-Dwamena C., Shu Q., Lin-Wang K., Zhang S., Wu J. (2019). The involvement of *PybZIPa* in light-induced anthocyanin accumulation via the activation of *PyUFGT* through binding to tandem G-boxes in its promoter. Hortic. Res..

[31] Bai S., Sun Y., Qian M., Yang F., Ni J., Tao R., Li L., Shu Q., Zhang D., Teng Y. (2017). Transcriptome analysis of bagging-treated red Chinese sand pear peels reveals light-responsive pathway functions in anthocyanin accumulation. Sci. Rep..

[32] Thomson G.E., Turpin S., Goodwin I. (2018). A review of preharvest anthocyanin development in full red and blush cultivars of European pear. N. Z. J. Crop. Hortic. Sci..

[33] Zhu Y.-F., Su J., Yao G.-F., Liu H.-N., Gu C., Qin M.-F., Bai B., Cai S.-S., Wang G.-M., Wang R.-Z. (2018). Different light-response patterns of coloration and related gene expression in red pears (*Pyrus* L.). Sci. Hortic..

[34] Sullivan J.A., Deng X.W. (2003). From seed to seed: The role of photoreceptors in *Arabidopsis* development. Dev. Biol..

[35] Banerjee R., Batschauer A. (2005). Plant blue-light receptors. Planta.

[36] Kinoshita T., Doi M., Suetsugu N., Kagawa T., Wada M., Shimazaki K. (2001). Phot1 and phot2 mediate blue light regulation of stomatal opening. Nature.

[37] Folta K.M., Pontin M.A., Karlin-Neumann G., Bottini R., Spalding E.P. (2003). Genomic and physiological studies of early cryptochrome 1 action demonstrate roles for auxin and gibberellin in the control of hypocotyl growth by blue light. Plant J..

[38] Wu S.H. (2014). Gene expression regulation in photomorphogenesis from the perspective of the central dogma. Annu. Rev. Plant Biol..

[39] Possart A., Xu T., Paik I., Hanke S., Keim S., Hermann H.-M., Wolf L., Hiss M., Becker C., Huq E. (2017). Characterization of phytochrome interacting factors from the moss *Physcomitrella patens* illustrates conservation of phytochrome signaling modules in land plants. Plant Cell.

[40] Imai H., Kawamura Y., Nagatani A., Uemura M. (2021). Effects of the blue light–cryptochrome system on the early process of cold acclimation of *Arabidopsis thaliana*. Environ. Exp. Bot..

[41] Bai S., Saito T., Honda C., Hatsuyama Y., Ito A., Moriguchi T. (2014). An apple B-box protein, MdCOL11, is involved in UV-B- and temperature-induced anthocyanin biosynthesis. Planta.

[42] Chen H., Xin G., Zhang B., Yang J.Y. (2009). Optimization of extraction technique of anthocyanin from red peel of ‘Nanguo’ pear. Food Sci..

[43] Wu J., Zhao G., Yang Y.-N., Le W.-Q., Khan M.A., Zhang S.-L., Gu C., Huang W.-J. (2013). Identification of differentially expressed genes related to coloration in red/green mutant pear (*Pyrus communis* L.). Tree Genet. Genomes.

[44] Huang C., Yu B., Teng Y., Su J., Shu Q., Cheng Z., Zeng L. (2009). Effects of fruit bagging on coloring and related physiology, and qualities of red Chinese sand pears during fruit maturation. Sci. Hortic..

[45] Li Y.-Y., Mao K., Zhao C., Zhao X.-Y., Zhang H.-L., Shu H.-R., Hao Y.-J. (2012). MdCOP1 ubiquitin E3 ligases interact with MdMYB1 to regulate light-induced anthocyanin biosynthesis and red fruit coloration in apple. Plant Physiol..

[46] Niu Q., Li J., Cai D., Qian M., Jia H., Bai S., Hussain S., Liu G., Teng Y., Zheng X. (2016). Dormancy-associated MADS-box genes and microRNAs jointly control dormancy transition in pear (*Pyrus pyrifolia* white pear group) flower bud. J. Exp. Bot..

