# Supplementary material for: Bagging Strategy and Identification of Coloring Mode of ‘Xinqihong’ Pear
Source: Int J Mol Sci. 2022 Jun 30;23(13):7310. doi: 10.3390/ijms23137310 (PMC9266653; doi:10.3390/ijms23137310)
Supplement: Supplementary file 1 [file ijms-23-07310-s001.zip › Fiugres.pdf]

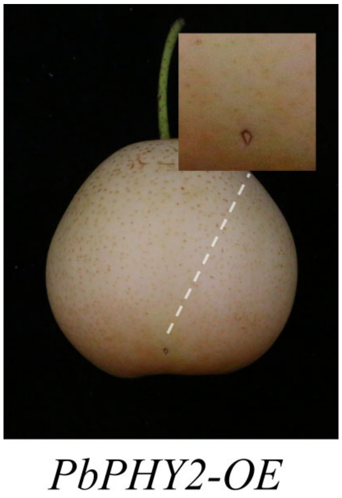

Figure S1. Overexpression verification of PbPHY2 in Pear peel.

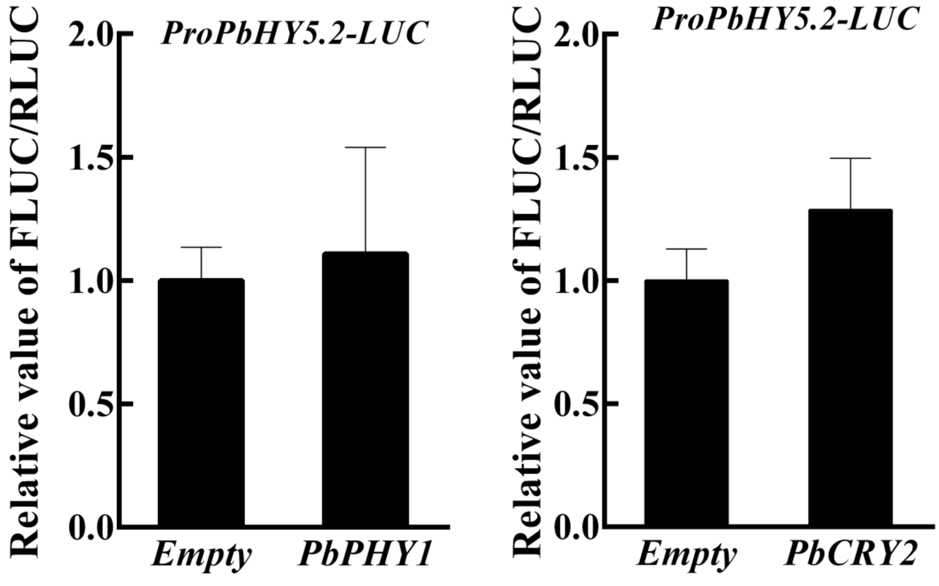

Figure S2. Transcriptional activation activity detection.
