# Supplementary material for: Bagging Strategy and Identification of Coloring Mode of ‘Xinqihong’ Pear
Source: Int J Mol Sci. 2022 Jun 30;23(13):7310. doi: 10.3390/ijms23137310 (PMC9266653; doi:10.3390/ijms23137310)
Supplement: Supplementary file 1 [file ijms-23-07310-s001.zip › Tables.pdf]

TableS1. Expression of genes related to anthocyanin, chlorophyll biosynthesis and light response in different color peel

Function	Term	Gene	FPKM		
			W	G	R
Synthesis	*PbCHS*	LOC103965055	43.11610	41.98108	457.24597
		LOC103945020	11.62585	12.00940	92.16743
		LOC103945021	0.26112	0.32838	5.72312
		LOC103959489	0.00000	0.00000	0.30846
	PbF3H	LOC103953484	16.07546	19.73626	110.23257
	PbUFGT	LOC103951514	1.25674	2.01201	10.71912
		LOC108865453	1.11588	0.50696	8.91425
		LOC103953250	3.31540	2.78618	6.42401
	PbDFR	LOC103954960	13.68957	16.82745	66.49716
		LOC103928717	5.66582	7.44067	29.76296
	PbANR	LOC103957850	26.67858	37.54422	56.89497
		LOC103929197	0.54301	0.40811	2.25619
		LOC103957769	27.82963	29.83311	21.90550
		LOC103937289	1.27367	2.24966	8.92135
	PbF3'H	LOC103930751	31.83941	33.26047	49.53490
	PbGST	LOC103959931	0.00000	0.13208	52.56742
		LOC103927795	10.48257	11.12437	31.49507
		LOC103938961	33.90763	29.94544	14.34464
		LOC103959477	22.89867	21.65967	10.53774
		LOC103960192	0.95482	1.70772	5.93781
		LOC103950893	3.23400	1.67022	10.55583
		LOC103945949	6.23930	8.07576	11.76673
		LOC103932093	22.14586	21.05315	12.54072
		LOC103927266	8.28757	7.95021	4.63588
		LOC103945951	0.10557	0.00000	1.24741
		LOC103945952	0.97151	0.23251	3.04357
	Pb4CL	LOC103951504	13.66886	5.72764	39.92463
		LOC103939486	1.51632	1.52271	4.68682
		LOC103929133	19.33696	19.09900	29.20429
		LOC103949908	3.78801	3.31282	5.59384
		LOC103943288	0.64540	0.96067	7.22157
	PbC4H	LOC103945990	348.50958	260.14399	163.89701
	PbANS	LOC103952863	4.02865	6.66786	77.68532
	PbCHI	LOC103940646	48.34979	58.44035	121.56509
	PbCHI	LOC103936753	15.29593	19.82761	43.21325

Synthesis	*PbHEMA*	LOC103956172	21.55181	19.41070	13.79175
		LOC103926826	9.22090	12.31961	7.37054
		LOC103942016	8.65978	11.11466	6.62633
		LOC103957838	2.64996	7.76462	4.58874
	PbGSA	LOC103936176	15.28846	18.98999	22.68677
		LOC103929993	12.45398	16.63593	14.52303
	PbHEMC	LOC103943220	16.76588	18.81135	21.25310
	PbHEMD	LOC103959538	11.86245	14.19072	15.29375
		LOC103957047	5.36536	5.70891	6.63630
	PbHEME	LOC103926881	14.74815	16.75848	18.73167
		LOC103953783	6.34935	10.53038	5.22621
		LOC103932108	3.81324	3.41149	3.57549
		LOC103953762	3.03575	0.61980	2.27299
		LOC103958685	0.21397	0.21727	0.14055
	PbHEMG	LOC103953066	13.87080	16.06470	10.50312
		LOC103960908	16.36129	14.57503	6.43179
		LOC103967516	11.44013	14.44300	10.27960
	PbCHLD	LOC103929545	9.02304	10.23033	15.26754
	PbCHLH	LOC103941422	22.90103	10.93832	8.40861
		LOC103933269	13.13644	6.70964	4.73153
		LOC103946503	7.17194	3.29611	3.87099
	PbCHLI	LOC103944127	43.66648	56.22799	38.39416
		LOC103939184	23.38292	27.09570	16.51518
	PbCHLM	LOC103931332	36.78969	44.34738	38.68050
	PbCRD1	LOC103938877	23.38442	18.97069	9.04117
		LOC103938880	17.03251	13.53193	6.89568
	PbDVR	LOC103958557	3.47942	5.42207	3.90045
		LOC103943616	3.70196	2.25865	3.48870
		LOC103937586	1.01214	0.57896	0.85057
		LOC103937579	0.10757	0.04277	0.07707
	PbCHLG	LOC103940196	7.80966	8.02849	3.47240
		LOC103958557	3.47942	5.42207	3.90045
		LOC103930527	4.69106	4.33636	2.69053
	PbCAO	LOC103940306	16.47997	10.43261	13.46249
Gene-Light	*PbPAL*	LOC103934948	19.73330	18.15927	60.97358
		LOC103962533	15.77197	20.76193	48.53800
	PbPIF	LOC103955840	64.93770	62.94662	35.45508
		LOC103955304	6.87206	7.03736	9.74329
		LOC103947396	0.42780	0.73335	0.06283
		LOC103928262	4.36709	3.82964	6.00392
	PbSPL	LOC103961786	23.71020	24.88857	36.69985

		LOC103936787	122.90023	97.29794	46.95872
		LOC103964035	7.41159	7.94851	13.65138
		LOC103958532	15.98956	16.85008	21.85343
		LOC103937943	11.99398	10.20364	17.79445
		LOC103947272	1.90458	1.09371	0.45925
		LOC103936162	10.97791	11.63708	17.84840
		LOC103952925	11.45555	12.17067	14.81884
		LOC103949991	1.44517	2.21930	0.41627
		LOC103944680	19.42836	19.30452	23.53102
		LOC103959155	11.79913	11.88534	2.28634
	PbPHY	LOC103930273	21.69145	20.32595	9.43001
		LOC103943526	3.23347	4.48445	8.18538
		LOC103950830	8.05150	8.37366	3.65124
		LOC103958720	2.29501	6.93558	0.66883
		LOC103951233	0.02879	0.01098	0.38024
	PbCRY	LOC103927183	21.96080	14.44676	21.06068
		LOC103967461	95.39634	98.50239	150.4083
		LOC103953562	9.27089	4.26801	4.40480
	PbPHOT	LOC103928382	50.18256	49.15203	106.6778
		LOC103928419	36.26618	32.24297	58.77983
		LOC108866695	33.22929	30.11649	45.34210
	PbUVR8	LOC103957087	66.20744	47.50742	34.65826
		LOC103947810	44.22825	48.80292	23.20890
		LOC103949855	49.64216	52.81717	65.45282
		LOC103962736	6.52929	5.99707	3.23185
		LOC103966963	27.46915	21.08760	15.33282
		LOC103935821	0.08456	0.53144	2.03180
		LOC103946833	30.06813	22.45127	24.83901
	PbCOP	LOC103948238	39.80696	50.18197	40.74700
		LOC103932349	40.68567	25.13941	23.00160
	PbHY5	LOC103946681	145.93442	31.20571	29.37038
		LOC103944715	40.14863	27.26435	48.15111
		LOC103940241	55.21004	12.95752	11.11171

Table S2. Fruit bag type

Bag types	Layer	Size/mm×mm	Color	Paraffin	Photo-permeability/%	Name code of treatments
Brown-Black bag	Double	160×210	Brown outside and black inside	No	0	BB-B
Yellow-White bag	Double	160×210	Yellow outside and white inside	No	24.66	YW-B
White bag	single	160×210	White	Yes	43.16	W-B
No bags covered	–	–	–	–	100	CK

Table S3. List of qRT-PCR Primers.

Primer used	Name	Forward primer(5'to3')	Reverse primer(3'to5')
For qPCR	*Actin*	ACAGTGTCTGGATTGGAGGGTC	CATTTGGAGAACTCAGAAGCACT
	PbC4H	AGCACACGGGCTACAACA	CAACGTGGTTTCAATAGCAGCAA
	PbCHS1	CCGTCGAGGAAGTTCGCAA	TCGGGATATGTGGCTTGATCC
	PbCHI1	ACCGAAGGGAAGCAAGAGTC	AGTTCAGCTGAGAGTGCGTT
	PbF3'H	ATGGTAGGACGGAGGCTCTT	GAATACTCCGGCCAACACCA
	PbDFR1	GGCCGCTACATTTGTTCGTC	TGCCCTTGAACTTTGTGGGT
	PbANR1	AGCAGTATTGGCCTTGCCAT	AAATATATGCGCCCGGCAGA
	PbANS	TGACCAGGCCTCTGGTAAGA	AAGTCACGCTTGTCCTCTGG
	PbUFGT1	TCGAAGAACTCGACCTCCCT	ATGGAGCGTCTTGCTTGTCA
	PbGST1	TTGAGGCAGGAGAGCACAAG	CCTGCATACTTGGCTGCGTA
	PbCRY1	GAGCAGGAGGTGTGGTTTGT	TTGGTGATGAGAGAAGTGCCA
	PbCRY2	CCCCTTGATGGGGTGTGAAT	TGTTCATCTTCCACGGCTCC
	PbPHY1	CGACTAGCGCGGTGAAGAAA	GATGGGACCAAGATCAGCCC
	PbPHY2	ACTGAAGCAACCTCTGTGCT	CCATTGATAATAACCGCCATCACC
	PbHY5.2	AGACGTTCCTTGGTTTTGGTCA	TTATTAGGAGGGGGCAACGC
For overexpression vector	*pGreenII 0029 62-SK-PbCRY1*	TAGAACTAGTGGATCCAGAGCAGGAGGT GTGGTTTG	CGGTATCGATAAGCTTTCACCCAG TTTGAGATAGCCGC
	pGreenII 0029 62-SK-PbCRY2	TAGAACTAGTGGATCCATGAGTGGCGAT AAGACTATAGTTTGGT	CGGTATCGATAAGCTTTCAGAGGT GTTCATCTTCCACGG
	pGreenII 0029 62-SK-PbPHY1	TAGAACTAGTGGATCCATGTCTTCTTCTA GACCTAGTGACTCATCAAG	CGGTATCGATAAGCTTTTACATCTT GTTTGCCACGGCG
	pGreenII 0029 62-SK-PbHY5.2	TAGAACTAGTGGATCCATGTCAGTCCCA ATCAGAGCAGG	CGGTATCGATAAGCTTTTACTTAA CTGATCCTTGCTTTTGCTCGA
For dual luciferase assay	*0800-proPbHY5.2*	CGGTATCGATAAGCTTCATGCAATGGTTT CAAAAAACTCGATAAGAATAAATATG	TAGAACTAGTGGATCCACACAATA TTAAGGAATATATATTGATTATATAT GCGCGTTTAGAT
	0800-proPbUFGT	CGGTATCGATAAGCTTGTTATATTGACAC AGGAGACACCGAG	TAGAACTAGTGGATCCGGTAGACG GAGAGCAAAGGCT
